# Growth Coordination Between Butyrate-Oxidizing Syntrophs and Hydrogenotrophic Methanogens

**DOI:** 10.3389/fmicb.2021.742531

**Published:** 2021-09-16

**Authors:** Shuqi Cong, Yiqin Xu, Yahai Lu

**Affiliations:** College of Urban and Environmental Sciences, Peking University, Beijing, China

**Keywords:** syntrophs, methanogens, growth synchronization, cell-to-cell interaction, microfluidic chip, butyrate

## Abstract

Syntrophy is a thermodynamically required mutualistic cooperation between fatty acid-oxidizing bacteria and methanogens that plays the important role in organic decomposition and methanogenesis in anoxic environments. In this study, three experiments were conducted to evaluate the cell-to-cell interaction in a thermophilic coculture consisting of *Syntrophothermus lipocalidus* and *Methanocella conradii* and a mesophilic coculture consisting of *Syntrophomonas wolfei* and *Methanococcus maripaludis*. First, syntrophs and methanogens were inoculated at different initial cell ratios to evaluate the growth synchronization. The quantitative PCR analysis revealed that the organism with a lower relative abundance at the beginning always grew faster, and the cell ratio converged over time to relative constant values in both the thermophilic and mesophilic cocultures. Next, intermittent ultrasound and constant shaking treatments were used to evaluate the influence of physical disturbance on microbial aggregation in the mesophilic coculture. The fluorescence *in situ* hybridization and scanning electron microscopy revealed that the tendency of syntrophic aggregation was not affected by the physical disturbances, although the activity was slightly depressed. *Syntrophomonas* dominated in the initial microbial aggregates, which, however, did not grow until *Methanococcus* was attached and increased to a significant extent, indicating the local growth synchronization during the formation and maturation of syntrophic aggregates. Last, microfluidic experiments revealed that whether or not *Syntrophomonas* or *Methanococcus* was loaded first, the second organism preferred moving to the place where the first organism was located, suggesting the cell-to-cell attraction between *Syntrophomonas* and *Methanococcus*. Collectively, our study demonstrated the growth synchronization and cell-to-cell attraction between the butyrate-oxidizing bacteria and methanogens for optimizing the syntrophic cooperation.

## Introduction

Microorganisms in nature rarely exist alone. For them to live together, certain cooperative rules must be established (D’Souza et al., 2018; Kassinger and van Hoek, 2020). One of the most remarkable types of microbial cooperation is the thermodynamically demanded syntrophy. In this relationship, one organism oxidizes a substrate, generating products, which are used by the second organism, and the latter consumes the products to a sufficiently low level so that the oxidation reaction, which is thermodynamically endergonic under standard conditions, becomes thermodynamically feasible. In this cooperation, the minimum energy from the initial substrate is shared by two organisms (Schink, 1997; Glissmann and Conrad, 2000; Sieber et al., 2012). Syntrophy occurs during anaerobic decomposition of organic matter in anoxic environments such as natural wetlands, lake sediments, rice paddy soils, and bioreactors; and it plays an important role in global carbon cycling and methane emissions. Though the ecological importance of microbial syntrophy has been well recognized, mechanisms underlying syntrophic partner cooperation remain poorly understood (Schink, 1997; McInerney et al., 2009; Muller et al., 2010).

Various strategies from molecular to community scales have been proposed for the syntrophic interaction between fatty acid-oxidizing bacteria and methanogens. These include the evolution of specific energy conservation mechanisms, selection of interspecies electron-transferring carriers, i.e., H_2_ versus formate, and the global regulation of transcriptional patterns under the conditions of syntrophic versus non-syntrophic growth (Stams and Plugge, 2009; Sieber et al., 2012; Krumholz et al., 2015; Schink et al., 2017; Liu and Lu, 2018). Recently, direct interspecies electron transfer is suggested for a few bacteria and methanogens to facilitate syntrophy (Lovley, 2017a, b). In all strategies, a fundamental principle is the spatial structuring of cell-to-cell interaction. Due to the thermodynamic constraints, the transfer of reducing equivalents (i.e., H_2_ and formate) from syntrophs to methanogens is considered to be the rate-limiting step of syntrophic methanogenesis (Schink, 1997; Schocke and Schink, 1997). The flux of H_2_ or formate between two microbial cells can be theoretically estimated according to Fick’s diffusion law, which states that the efficiency of interspecies hydrogen or formate transfer is negatively correlated with the interspecies cell-to-cell distance (Scholten and Conrad, 2000). Consequently, the close proximity between syntrophs and methanogens is essential for the efficient syntrophy.

Microbial aggregates such as granules and biofilms are ubiquitous in environments, which can facilitate the resistance of microbial community to environmental stresses such as antibiotics and the limitation of nutrients (Flemming and Wingender, 2010). In methanogenic communities, e.g., those from natural environments (Zhang and Lu, 2016; Zhang et al., 2018) and waste water treatment systems (Skiadas et al., 2003), fluorescence *in situ* microscopy has revealed that syntrophs are in close proximity to methanogens, forming microbial aggregates (Imachi et al., 2000). Co-aggregation was detected in defined cocultures of syntrophs and methanogens that otherwise do not form aggregates in pure cultures (Ishii et al., 2005). Chemotaxis and signal transduction such as quorum sensing may direct the formation of microbial aggregates or biofilms (Shih and Huang, 2002; Shrout and Nerenberg, 2012). It has been suggested that the flagellar proteins produced by *Pelotomaculum thermopropionicum*, a propionate-oxidizing syntroph, were bound to the cell surfaces of *Methanothermobacter thermautotrophicus* and stimulated methanogenesis-related gene expression (Shimoyama et al., 2009). Collectively, to develop syntrophic interactions, syntrophs and methanogens may employ specific mechanisms for interacting with their partners and coordinating their syntrophic growth. In the present study, we aimed to investigate (i) how syntrophic bacteria and methanogens synchronized their growth and (ii) whether active attraction occurred between syntrophic bacteria and methanogens to form microbial aggregates. The butyrate-oxidizing syntrophs, *Syntrophomonas wolfei* and *Syntrophothermus lipocalidus*, and the hydrogenotrophic methanogens, *Methanococcus maripaludis* and *Methanocella conradii*, were used to construct two defined, thermophilic and mesophilic cocultures; and batch and microfluidic incubations were performed to characterize their syntrophic interactions.

## Materials and Methods

### Cultivation of Pure Cultures

*Syntrophomonas wolfei* G311 (DSM102351), *S. lipocalidus* TGB-C1 (DSM12680), and *M. maripaludis* S2 (DSM14266) were purchased from German culture collection DSMZ (Braunschweig, Germany). *M. conradii* strain HZ254^*T*^ was isolated and was routinely maintained in our lab as described previously (Lu and Lu, 2012). The pure cultures of *S. wolfei* and *S. lipocalidus* were cultivated in medium containing 20 mM of sodium crotonate as described (Liu et al., 2014; Zhang et al., 2018). *M. maripaludis* was cultivated in a modified DSMZ141 medium containing 100 mM of NaCl, 7.87 mM of MgCl_2_⋅6H_2_O, and 0.007 mM of Fe(NH_4_)_2_(SO_4_)_2_ under 170 kPa of H_2_/CO_2_ (80:20, v/v) (Zhang et al., 2018).

### Batch Incubation of the Defined Cocultures

Two defined cocultures, one pairing *S. wolfei* with *M. maripaludis* (hereafter refer to the WM coculture, which is mesophilic) and the other pairing *S. lipocalidus* with *M. conradii* (hereafter refer to the LC coculture, which is thermophilic), were constructed. The cocultures were prepared by mixing different volumes of the log phase *S. wolfei* or *S. lipocalidus* with the vigorously growing *M. maripaludis* or *M. conradii* in the medium of the respective methanogens. The headspace of 250-ml incubation bottles was flushed with N_2_ for 5 min and then adjusted with N_2_:CO_2_ (80:20 [v/v]) to a final pressure of 172 kPa. All the bottles were sealed with butyl stoppers and crimped aluminum caps. Cultivation was carried out at 35°C for the mesophilic WM coculture and at 55°C for the thermophilic LC coculture under dark without agitation. The sodium butyrate was added as sole substrate to a final concentration of 10 mM. To create the treatments for different start conditions, the volumes of the syntrophs and methanogens for inoculation were adjusted so that high, medium, and low ratios of syntroph versus methanogen cells were established at the beginning. The exact ratios were different between the two defined cocultures, which was based on the pre-experiment growth of the cocultures.

### Chemical Analysis

Gasses were sampled with a Pressure-Lok syringe (VICI, Houston, TX, United States); and the concentration of methane and hydrogen was determined using a gas chromatograph (7890A, Agilent, Santa Clara, CA, United States) equipped with an 80/100-mesh Porapak Q column (Supelco; Sigma-Aldrich, St. Louis, MO, United States) and a thermal conductivity detector (Liu et al., 2014). The unit of CH_4_ concentration was converted from partial pressure in headspace to mmol L^–1^ in liquid medium by using Avogadro’s law. Liquid samples (0.5 ml) were collected periodically with a sterile syringe, centrifuged, and filtered through 0.22-μm filters. Concentrations of butyrate and acetate in culture medium were determined by high-performance liquid chromatography with a ZORBAX SB-Aq C18 organic acid column (250 by 4.6 mm; particle size 5 μm; Agilent) at a flow rate of 0.8 ml min^–1^. The UV absorbance detector was set at 210 nm (Zhang et al., 2018).

### Quantitative PCR Analysis

qPCR was used to estimate the growth of syntrophs and methanogens during incubation. Cells were harvested periodically from the syntrophic cocultures. The cell suspensions were centrifuged at 20,817 × *g* at 4°C for 8–10 min (Avanti J-26XP, Beckman Coulter, Brea, CA, United States). The DNeasy^®^ Blood & Tissue kit (Qiagen, Hilden, Germany) was used to extract microbial DNA from the pellets following the procedure of the manufacturer. The purified DNA samples were stored in 50 μl of Buffer AE at –20°C. The qPCR was performed using ABI Prism 7500 Real-time qPCR Detection System (Applied Biosystems, Foster City, CA, United States) with the primer pairs of Arc364r/915r for methanogens and Bac338f/518r for syntrophs. The PCR mixture of 20 μl contained 10 μl of PowerUp SYBR Green PCR Master Mix (Applied Biosystems), 0.8 μl of each primer (stock concentration of 10 μM), 1 μl of template DNA, and 7.4 μl of ddH_2_O. Each measurement was performed in triplicate. The thermal cycles and fluorescence signal acquisition followed the protocols as described previously (Ma et al., 2013; Hu and Lu, 2015). Data analysis was carried out with 7500 System SDS Software. Standard curves were obtained using serial dilutions (10^8^–10^2^ copies μl) of linearized plasmids containing cloned fragments of either bacterial 16S rRNA or archaeal 16S rRNA (Liu and Lu, 2018). The cloned fragments were obtained through PCR from pure culture DNA using the primer pairs described above. One-way ANOVA and Duncan’s *post hoc* test was used for testing significant differences in qPCR data using SPSS software (version 17).

### Microscopy

Fluorescence *in situ* hybridization (FISH) analysis was performed for the WM coculture on 4% paraformaldehyde-fixed samples according to a procedure described previously (Moter and Gobel, 2000). Oligonucleotide probes specific for bacteria (Cy3-labeled EUB338mix probes) and archaea (fluorescein isothiocyanate (FITC)-labeled ARC915 probe) were used. The details of the probes design are available in the probeBase (Loy et al., 2003). The labeled samples were visualized using epifluorescence microscopy (Axioimager D2, ZEISS, Oberkochen, Germany) (Zhang and Lu, 2016). The brightness of fluorescence images was optimized by ImageJ (Ciniselli et al., 2015). The cells in the lag phase, early exponential phase, mid-log phase, and late exponential phase were collected and observed. For scanning electron microscopy (SEM), cells were collected by a syringe, fixed with 2.5% (wt/vol) glutaraldehyde in phosphate-buffered saline, and sequentially dehydrated with ethanol [20, 40, 60, 80, 95, and 100% (v/v) ethanol, 10 min for each step]. The dried samples were coated with platinum and imaged using SEM (Axio imager D2, ZEISS) (Zhang and Lu, 2016).

### Microfluidic Chip Experiment

The microfluidic experiment was conducted for the WM coculture. The microfluidic chips were fabricated following the protocol as described (Sun et al., 2016). A silicon substrate (100 mm) was heated at 190°C for 5 min. The positive lift-off photoresist SU8 3050 (MicroChem Corp, Newton, MA, United States) was sequentially deposited and was exposed to the photomask at a UV light. Then, the lift-off process was applied to remove the photoresist. The microfluidic channels were made using soft lithographic techniques. The elastoplastic material polydimethylsiloxane (PDMS) (RTV615, Momentive, Waterford, NY, United States) mixture was degassed and poured into the SU8 molds (Sun et al., 2016). The thickness of the PDMS was 170 μm. After the PDMS replica was cured and released, the cleaned glass microfluidic substrate and PDMS replica were surface treated by air plasma in a reactive ion etching system. Immediately, the PDMS replica was placed against the glass substrate. The clamped PDMS replica and glass substrate were subsequently placed in an oven at 70°C for 1 h.

Three batches of microfluidic experiments were conducted. The microfluidic platform was placed within an anaerobic glove box to maintain an anaerobic condition for all experiments. First, the log-phase *S. wolfei* in pure culture was pumped into the channels of the chip at the speed of 6,000 μl h^–1^ until the channels were fully filled with the culture (Supplementary Figure 1). The entire chip was then tilted to make the cells move into the wells at one side of the chamber under gravity. Two hours later, the chip was placed back horizontally. The remaining *S. wolfei* in channel passages were washed out thoroughly with the fresh medium at the speed of 200 μl h^–1^. Then the *M. maripaludis* were pumped into channels at the speed of 200 μl h^–1^ for 2 h. Finally, the velocity of mass flow in channels was reduced to 10 μl h^–1^ for maintaining the liquid environment in chip while reducing the disturbance of liquid flow for cell cultivation. For the second batch experiment, similar procedure was conducted except the reverse of order for cell injection; i.e., *M. maripaludis* was injected first and then followed by *S. wolfei*. The third batch was a single organism control experiment where only the *S. wolfei* cells were used in both steps of cell injection.

## Results

### Coordinated Growth of Syntrophs and Methanogens

**FIGURE 1:**
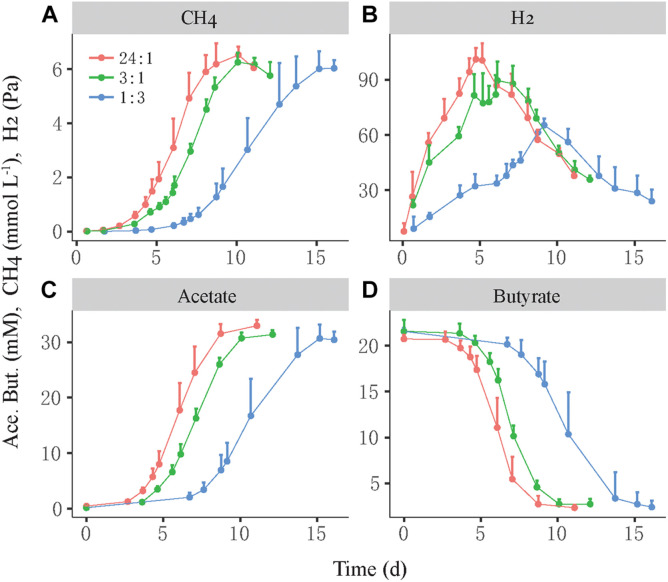
Syntrophic oxidation of butyrate by a thermophilic coculture consisting of *Syntrophothermus lipocalidus* and *Methanocella conradii*. The production of CH_4_ **(A)**, H_2_ **(B)**, and acetate **(C)** and the consumption of butyrate **(D)** are shown. The actively growing *S. lipocalidus* and *M. conradii* in pure cultures were inoculated to fresh medium with different population sizes so that three levels of syntroph-to-methanogen cell ratios (i.e., 24:1, 3:1, and 1:3) were created at the beginning, which were designated as the high, middle, and low cell ratio treatments, respectively. The error bars indicate the positive values of standard deviation of four replicates.

We investigated the growth synchronization between butyrate-oxidizing syntrophs and methanogens by setting different initial ratios of two populations in the coculture. The synchronization between *S. lipocalidus* and *M. conradii* was tested by setting the initial cell ratio of *S. lipocalidus* versus *M. conradii* to 24:1, 3:1, and 1:3, which were designated as the high, middle, and low ratios, respectively. The high ratio showed the shortest lag phase of CH_4_ production followed by the middle ratio, whereas the lag phase nearly doubled in the low-ratio treatment (Figure 1A). Moreover, butyrate consumption and acetate accumulation were faster for the high and middle ratios than the low-ratio treatment (Figures 1B,D). H_2_ accumulated transiently, with the maximal concentration being higher and earlier in the high- and middle-ratio treatments compared with the low-ratio treatment (Figure 1C).

**FIGURE 2:**
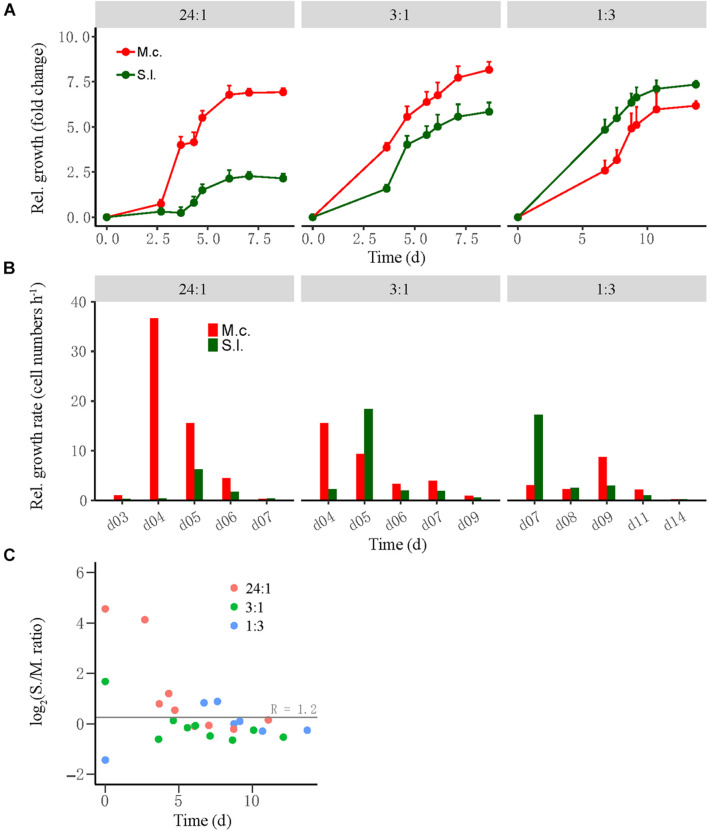
The growth synchronization of *Syntrophothermus lipocalidus* (S.l.) and *Methanocella conradii* (M.c.) in the thermophilic coculture. The relative growth **(A)** of the individual populations was estimated as the fold change of cell number at any timepoint relative to that at the beginning, while the relative growth rate **(B)** was defined as the difference in cell number between two timepoints divided by the duration. Note that the change in *Syntrophothermus*–*Methanocella* cell ratio over time **(C)** is shown in logarithmic scale. The error bars in **(A)** indicate the positive values of standard deviation of four replicates.

Cocultures were evaluated by the relative growth and relative growth rate of each partner as determined by qPCR of subsamples collected during incubation. The relative growth was defined as the fold change of cell number at time T relative to that at time 0, while the relative growth rate was defined as the difference in cell number between time T2 and time T1 divided by the duration between two timepoints. For the high-ratio treatment where butyrate oxidation was more rapid, the cell number of *M. conradii* increased 4.5-fold in 3–4 days and 7.5-fold in 6 days and then leveled off, whereas the cell number of *S. lipocalidus* increased only 2.5-fold at day 6 and leveled off (Figure 2A, left). The relative growth rate of *M. conradii* reached the maximum of 38 cell numbers h^–1^ at day 4; meanwhile, that of *S. lipocalidus* remained close to zero (Figure 2B, left). For the middle-ratio treatment, the relative growth displayed a similar pace for two populations, but reaching greater values for *M. conradii* than for *S. lipocalidus* (Figure 2A, middle). Importantly, the relative growth rate showed a shift from being significantly greater for *M. conradii* at day 4 to being significantly greater for *S. lipocalidus* at day 5 (Figure 2B, middle). For the low-ratio treatment, both the relative growth and the relative growth rate of *S. lipocalidus* significantly surpassed those of *M. conradii*. Only in the later stage (after 9 days) did the relative growth rate of *M. conradii* become greater than the *S. lipocalidus* (Figures 2A,B, right). We calculated the cell ratio (*R*) of *S. lipocalidus* versus *M. conradii* over the incubation, which showed that the log_2_*R* value fluctuated in the early stage depending on the initial ratio treatment but finally converged at about *R* = 1.2 from day 5 forward for all the three treatments (Figure 2C). The total biomass and the Gibbs free energy changes available for *S. lipocalidus* and *M. conradii* are illustrated in Supplementary Figures 2, 3. The Gibbs free energy changes showed no significant difference among the cell ratio treatments, albeit the shift in timeframe of availability (Supplementary Figure 3). The highest biomass accumulation was detected in the middle-ratio treatment followed by the high-ratio treatment, while only about a half of biomass was formed in the low-ratio treatment compared with the middle ratio (Supplementary Figure 2).

**FIGURE 3:**
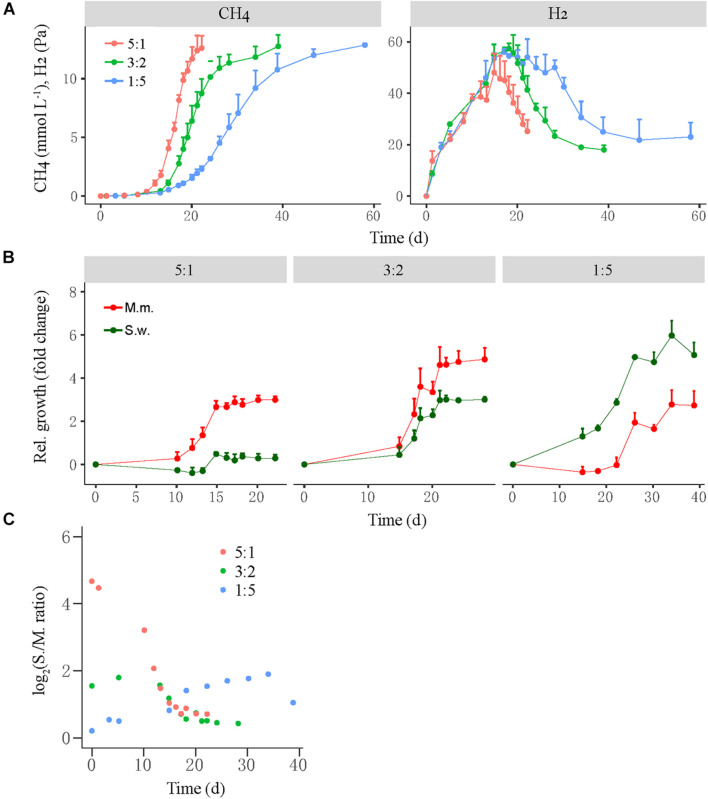
The production of CH_4_ and H_2_ from butyrate oxidation **(A)** by a mesophilic coculture consisting of *Syntrophomonas wolfei* (S.w.) and *Methanococcus maripaludis* (M.m.) and their growth synchronization **(B,C)**. Three levels of syntroph-to-methanogen cell ratios (i.e., 5:1, 3:2, and 1:5) were created at the beginning by inoculating different population sizes to fresh medium, which were designated as the high, middle, and low cell ratio treatments, respectively. The relative growth **(B)** of the individual populations was estimated as the fold change of cell number at any timepoint relative to that at the beginning. Note that the change in *Syntrophomonas*–*Methanococcus* cell ratio over time **(C)** is shown in normal scale. The error bars in **(A,B)** indicate the positive values of standard deviation of four replicates.

A similar test was conducted for the mesophilic WM coculture, in which the initial cell ratio of *S. wolfei* versus *M. maripaludis* was set at 5:1, 3:2, and 1:5 for the high-, middle-, and low-ratio treatments, respectively (Figure 3). Like in the thermophilic LC coculture, the high-ratio treatment showed the shortest lag period of CH_4_ production (10–12 days) followed by the middle-ratio treatment (15 days), while the low-ratio treatment had the longest lag phase (18–20 days) (Figure 3A, left). The transient H_2_ accumulation in three treatments was correlated with the CH_4_ production (Figure 3A, right). The growth of *M. maripaludis* initiated at day 10, increased by three-fold at day 15 and then leveled off (Figure 3B, left), while *S. wolfei* exhibited only minor growth at day 15. For the middle-ratio treatment, the growth of both *M. maripaludis* and *S. wolfei* initiated at day 15, displaying moderately greater values for *M. maripaludis* than for *S. wolfei* (Figure 3B, middle). By comparison, the relative growth was much greater for *S. wolfei* than for *M. maripaludis* in the low-ratio treatment (Figure 3B, right). Similar to the LC coculture, the cell ratio of the WM cocultures varied in the early stage but converged at around 1 from day 15 forward for all the three treatments.

### Formation and Activity of Syntrophic Aggregates

**FIGURE 4:**
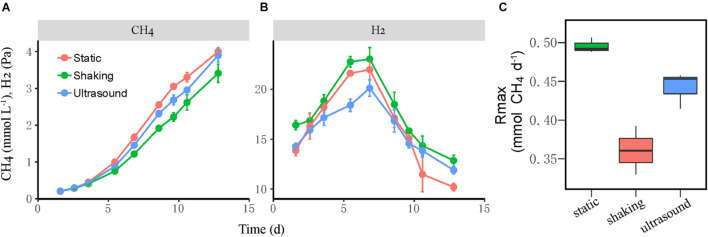
Test for the influence of physical disturbance on the activity of the mesophilic *Syntrophomonas*–*Methanococcus* coculture. The actively growing coculture was inoculated to fresh medium, and two disturbance treatments, i.e., intermittent ultrasound and constant shaking, were applied with the static incubation served as the control. Shown are the production of CH_4_ **(A)** and H_2_ **(B)** from butyrate oxidation in three parallel incubations. The maximal rate of CH_4_ production **(C)** was calculated during the period of linear increase of CH_4_ concentration. The error bars indicate the standard deviation of four replicates.

Next, we tested the effect of physical disturbance on the formation and activity of microbial aggregates in the mesophilic WM coculture. The actively growing coculture was inoculated to fresh medium; and two disturbance treatments including the intermittent ultrasound and constant shaking (180 r min^–1^) of the incubation bottles were applied, with static incubation serving as the control. The new cocultures presumably had the initial syntroph-to-methanogen cell ratio inherited from the inoculants. The results showed that the activity of CH_4_ production was slightly repressed by both ultrasound and shaking treatments compared with the static control (Figure 4A). We calculated the maximum rate of CH_4_ production during the periods when CH_4_ concentration linearly increased in headspace, which revealed 10% reduction in the ultrasound and 25% reduction in the constant shaking treatment compared with the static control (Figure 4C). H_2_ accumulated transiently to a concentration of 20–25 Pa with the higher values for the shaking treatment (Figure 4B).

**FIGURE 5:**
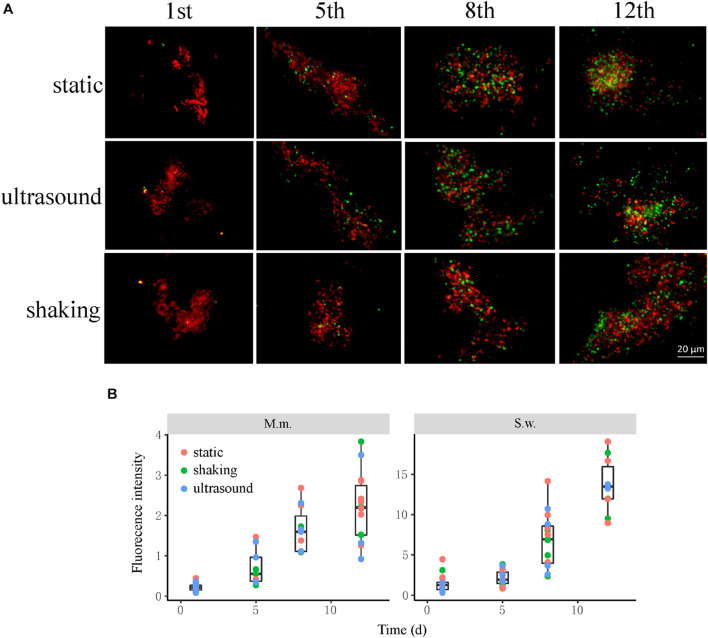
The influence of physical disturbance on the formation and maturation of syntrophic aggregates in the *Syntrophomonas*–*Methanococcus* coculture. Fluorescence *in situ* hybridization (FISH) observations **(A)** were made at different timepoints over the incubation. Oligonucleotide probes specific for bacteria (Cy3-labeled EUB338mix probes in red) and archaea (fluorescein isothiocyanate (FITC)-labeled ARC915 probe in green) were used. Shown are the representative images of more than 10 observations in each sample. The box plots **(B)** shown are the fluorescence intensity of the individual populations estimated using the ImageJ software. The center line denotes the median, the boxes cover the 25th and 75th percentiles, and the whiskers extend to the all data points. See Figure 3 for more explanation about the incubation and treatments.

**FIGURE 6:**
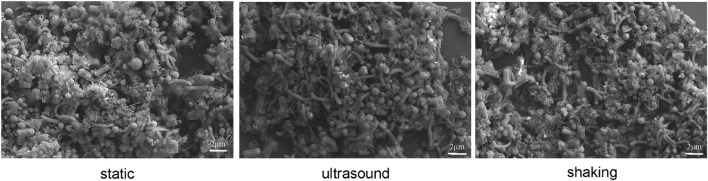
Scanning electron micrographs of cell aggregates in *Syntrophomonas*–*Methanococcus* coculture at the end of incubation. Intermittent ultrasound and constant shaking were applied as the physical disturbance treatments with the static condition as the control.

Culture samples were collected four times from incubations for FISH observation. Microbial aggregates were detected at the first day, which were composed mainly of the *S. wolfei* cells (Figure 5A). At the fifth day, corresponding to the initiation of coculture growth (Figure 4A), the cells of *M. maripaludis* gradually increased surrounding the aggregates. At the eighth day, corresponding to the mid-exponential phase, the size of aggregates significantly enlarged with the increased cell numbers of both *S. wolfei* and *M. maripaludis* within aggregates. Further growth of syntrophic aggregates was observed at the 12th day in the stationary phase when most of butyrate was already consumed. The syntroph and methanogen cells were found interconnected densely within the mature aggregates (the eighth and 12th days in Figure 5). Notably, there was no significant difference in the tendency of microbial aggregation among the three treatments. The fluorescence intensity of the individual populations was estimated using the ImageJ software, which indicated a nearly linear increase of *M. maripaludis* cells over 2 weeks’ incubation (Figure 5B, left), while *S. wolfei* showed an obvious lag phase during the initial 5 days (Figure 5B, right). Consistent with the pattern of FISH images, the fluorescence intensity of two populations showed no significant difference among the three treatments. SEM observation made at the end of incubations revealed the extensive formation of extracellular polymeric substances (EPSs) that physically served as the matrix support for microbial aggregation (Figure 6). The influence of disturbance treatments, however, could not be explicitly identified in SEM images. These results suggest that the *S. wolfei* cells initiated the syntrophic aggregation and allowed for the attachment and gradual growth of *M. maripaludis* before the rapid activity of syntrophic coculture. Importantly, although the activity of the coculture was influenced by two disturbance treatments, the tendency of microbial aggregation was not.

### Cell-to-Cell Attraction Revealed by Microfluidic Incubation

Last, we performed the microfluidic chip experiments to observe the cell-to-cell interaction in the WM coculture. The chip contained two symmetric wells for culture incubation along the opposite sides of flow channels (Supplementary Figure 1). The flow medium contained 10 mM of butyrate and was used throughout all microfluidic experiments. Three alternate tests were conducted. First, *S. wolfei* cells were injected; and the chips were tilted to allow more cells shunted to the left-side well than the right-side well. The chips were then returned to horizontal position; and after the remaining cells were washed thoroughly away from chip channels, *M. maripaludis* cells were loaded for 2 h and further incubated for over 20 h. We found that more of *M. maripaludis* cells entered the left-side well than the right-side (Supplementary Video 1). Over the 20-h incubation, the coculture in the left-side well grew to a much larger population than in the right-side well. In the next test, we changed the order of cell loading so that the *M. maripaludis* cells were injected first with more cells loaded to the left-side well than the right-side. The following feeding of *S. wolfei* cells revealed that more of *S. wolfei* cells entered the left-side well where more of the *M. maripaludis* cells were already located (Supplementary Video 2); and the populations increased to a much greater extent in the left-side well. The third test was used to verify if the inertia of microfluidic flow influenced the cell movement. Here, we pumped the *S. wolfei* first and tilted the chip to the right side so that more *S. wolfei* cells were loaded to the right-side well at the beginning. After returning the chip to horizontal position, we injected the *S. wolfei* cells again. Within 2 h of the cell injection, we observed that the *S. wolfei* cells entered the wells of both sides without a preference (Supplementary Video 3). Further incubation was not continued as *S. wolfei* could not grow on butyrate alone in the media.

## Discussion

Microbial aggregation reduces the cell-to-cell distance for interspecies H_2_ or formate transfers and hence facilitates syntrophic interactions between fatty acid-oxidizing bacteria and methanogens involved in the decomposition of organic matter and CH_4_ emissions in anoxic environments (Boone et al., 1989; Ishii et al., 2006; Stams and Plugge, 2009). How syntrophs and methanogens coordinate the cell-to-cell interaction, however, remains poorly understood. Here, we show that the butyrate-oxidizing bacteria and methanogens when cocultured together displayed growth synchronization and cell-to-cell attraction for the syntrophic interaction.

We tracked the relative growth of butyrate-oxidizing bacteria versus methanogens in two different cocultures. The thermophilic coculture consisted of *S. lipocalidus* and *M. conradii*; and the mesophilic coculture comprised *S. wolfei* and *M. maripaludis*. Probably owing to the thermodynamic advantage, the growth of the thermophilic LC coculture was faster than that of the mesophilic WM coculture (Figures 1, 3). To evaluate the growth synchronization, three levels of the initial cell ratio were prepared to create different population sizes of syntrophs versus methanogens in cocultures at the beginning. We found that the activity of both the thermophilic and mesophilic cocultures benefited from a high cell ratio of syntrophs versus methanogens, and a better biomass accumulation was observed in the thermophilic coculture with the middle-ratio treatment (Supplementary Figures 2, 3). These results reflect the primary role of bacteria in the initiation of syntrophic interaction, which provides the substrate and energy source for the methanogen activity. Interestingly, in both the thermophilic and mesophilic cocultures, the high initial ratio was associated with a faster and greater relative growth of methanogens, while the low initial ratio led to the greater relative growth of syntrophs (Figures 2, 3). Eventually, the syntroph-to-methanogen cell ratio converged at a relatively constant value regardless of the initial ratio treatments. Apparently, the relative growth of syntrophs versus methanogens in the cocultures was regulated by the initial cell ratio, ending up at an optimized cell ratio for the syntrophic interaction. These results demonstrated the growth synchronization between the butyrate-oxidizing syntrophs and methanogens in the cocultures. A few previous studies also indicated that the syntrophically growing cocultures of *Desulfovibrio vulgaris* and *M. maripaludis* maintained a relatively constant cell ratio during the steady-state growth (Stolyar et al., 2007; Walker et al., 2009).

We then observed the formation of syntrophic aggregates in the WM coculture. The ultrasound and shaking treatments were applied to evaluate the influence of physical disturbance on the microbial aggregation. FISH images revealed that the initial aggregates consisted mainly of *Syntrophomonas* cells (Figure 5A). These cells, however, did not grow until *Methanococcus* cells were attached and increased to a significant extent. Since inoculants were collected from the actively growing WM coculture, the initial syntroph vs. methanogen cell ratio was presumably optimized. Thus, our FISH observation suggested that although the initially optimized cell ratio, the formation and maturation of syntrophic aggregates required local growth synchronization between *Syntrophomonas* and *Methanococcus*. More importantly, this pattern of microbial aggregation was not affected by the physical disturbances, although a slight decrease in activity was observed (Figure 5 vs. Figure 4). The robustness of syntrophic aggregates was further supported by SEM observation, which showed the production of large amount of EPS (Figure 6) that served as the matrix substrate for microbial aggregation (Adav et al., 2008).

Last, we performed microfluidic experiments to evaluate the cell-to-cell interaction in the WM coculture. The laminar flow in microfluidic system has the advantage of preventing environmental interference on cell movement. The microfluidic chip used in the present experiment had two symmetric wells along the media flow channel (Supplementary Figure 1). Cells would enter the wells of both sides randomly and equally under the horizontal condition without pretreatment to chip system. For the tests, we loaded the cells individually, and chip system was managed so that one of the syntrophic partner (either *Syntrophomonas* or *Methanococcus*) was loaded to the well of one side before the loading of the second organism. We found that regardless of whether *Syntrophomonas* or *Methanococcus* was loaded first, the second organism preferred moving to the well where the first organism was already located (Supplementary Videos 1, 2). Such preference of the cell movement was not detected in the control test with the loading of single identical organism, i.e., *Syntrophomonas* (Supplementary Video 3). Therefore, we assumed that specific cell-to-cell attraction occurred between *Syntrophomonas* and *Methanococcus* under the microfluidic conditions. The mechanisms remain unclear. Many microorganisms in environments employ flagella and chemotaxis to locate the favorable niches (Naether et al., 2006; Porter et al., 2011). Other microbes have evolved specific signal transduction system, such as quorum sensing, for cell-to-cell communication (Zhu and Mekalanos, 2003). For instance, it has been shown that the propionate-oxidizing *P. thermopropionicum* utilizes flagellar proteins to develop specific communication with *M. thermautotrophicus* for syntrophic propionate oxidation and methanogenesis (Shimoyama et al., 2009). Both *S. wolfei* and *M. maripaludis* have the complete sets of flagella and chemotaxis genes in their genomes (Hendrickson et al., 2004; Sieber et al., 2010). These flagella can be observed by transmission electron microscopy. A few studies have shown that flagella are required for swimming and efficient surface attachment in both organisms (Cutter et al., 2003; VanDyke et al., 2009; Jarrell et al., 2011). However, it remains unclear whether these structures and communication mechanisms work in their syntrophic cocultures (Krumholz et al., 2015). More work is needed to elucidate the mechanisms for cell-to-cell interaction in different syntrophic partnerships.

In summary, we show in this study that the butyrate-oxidizing syntrophs and hydrogenotrophic methanogens reveal the growth synchronization and cell-to-cell attraction. These ecological processes can greatly facilitate their syntrophic metabolisms and especially the formation of syntrophic aggregates. A few hypotheses have been proposed for the aggregation of anaerobic organisms (Jiang et al., 2015). For instances, Ishii and colleagues have proposed that *P. thermopropionicum* filaments play an important role in microbial aggregation (Shimoyama et al., 2009). Another idea is the “Cape Town hypothesis,” which proposes the involvement of extracellular polypeptides in the formation of anammox granules (Lu et al., 2012). Our study suggests that specific cell-to-cell attraction occurs between butyrate-oxidizing syntrophs and methanogens, which may facilitate their aggregation and growth synchronization for optimizing the syntrophic cooperation. Further research is warranted to elucidate the molecular mechanisms and to determine if these ecophysiological processes happen in other syntrophic bacteria.

## Data Availability Statement

The original contributions presented in the study are included in the article/Supplementary Material, further inquiries can be directed to the corresponding author.

## Author Contributions

YL, SC, and YX contributed to design of the study and performed the experiments. YL and SC performed the data analysis and wrote the first draft of the manuscript. All authors contributed to manuscript revision, read, and approved the submitted version.

## Conflict of Interest

The authors declare that the research was conducted in the absence of any commercial or financial relationships that could be construed as a potential conflict of interest.

## Publisher’s Note

All claims expressed in this article are solely those of the authors and do not necessarily represent those of their affiliated organizations, or those of the publisher, the editors and the reviewers. Any product that may be evaluated in this article, or claim that may be made by its manufacturer, is not guaranteed or endorsed by the publisher.
