# Supplementary material for: Growth Coordination Between Butyrate-Oxidizing Syntrophs and Hydrogenotrophic Methanogens
Source: Front Microbiol. 2021 Sep 16;12:742531. doi: 10.3389/fmicb.2021.742531 (PMC8481629; doi:10.3389/fmicb.2021.742531)
Supplement: Supplementary file 4 [file Table_1.DOCX]

**Supporting information**

**Growth coordination between butyrate-oxidizing syntrophs and hydrogenotrophic methanogens**

Shuqi Cong^1^, Yiqin Xu^1^ and Yahai Lu^1*^

^1^College of Urban and Environmental Science, Peking University, Beijing, 100871, China

^*^To whom correspondence should be sent:

Yahai Lu, College of Urban and Environmental Science,

Peking University,

Yiheyuan Road 5, Beijing, China

Phone/Fax: +86 10 62750669

E-mail: luyh@pku.edu.cn

**Legends to Supplementary Figures and Videos**

**Figure S1:** Microfluidic chip and experimental setup. (A) overview of chip system with the coin shown for the comparison of chip size; (B) a schematics of the device. The device was composed of four identical units. There are two inflows and one outflow to each unit; (C) experimental setup showing (a) the loading of the first organism, (b) tilting the chip so that more cells were located to the one side wells, (c) washing out the remaining cells in the chip channels, and (d) loading of the second organism.

**Figure S2:** The total biomass production of a thermophilic coculture consisting of *Syntrophothermus lipocalidus* and *Methanocella conradii* estimated by qPCR of subsamples. The actively growing *S. lipocalidus* and *M. conradii* in pure cultures were inoculated to fresh medium with different population sizes so that three levels of syntroph-to-methanogen cell ratios (*R*) were created at the beginning for cocultures. Biomass_A, *R*=24:1; Biomass_B, *R*=3:1; Biomass_C, *R*=1:3. The error bars indicate the positive values of standard deviation of four replicates. The data indicate the net accumulation of biomass from three different treatments.

**Figure S3:** Gibbs free energy change available for *Syntrophothermus lipocalidus* and *Methanocella conradii* during their syntrophic growth on butyrate with different treatments of the initial cell ratio (R). Gibbs free energies (ΔG) of the individual reactions were calculated from the standard Gibbs free energies (ΔG^o^) and the actual concentrations of reactants and products as described previously (Rothfuss and Conrad, 1993).The standard Gibbs free energies (ΔG^o^) of the reactions were calculated from the standard Gibbs free energies of formation (G_f_^o^) of the reactants and products using literature data (Thauer et al., 1977). A, R=24:1; B, R=3:1; C, R=1:3. Closed and open symbols indicate the Gibbs free energies of the butyrate-oxidizing and H_2_-producing reaction (H_2_) and the H_2_-oxidiang and CH_4_-producing reaction (CH_4_), respectively.

**Supplementary Video 1.** Microfluidic experiment showing the movement of *Methanococcus* *maripaludis* and the growth of the Syntrophomonas-Methanococcus coculture. *Syntrophomonas wolfei* was injected first with more cells loaded to the left well though tilting the chip system. After returning chip to the horizontal position, Methanococcus cells were injected and the coculture was further incubated for over 20 hours in microfluidic system.

**Supplementary Video 2.** This was basically the same experiment as shown in Video 1 except the change of order for cell loading. Here the *Methanococcus maripaludis* cells were injected first with more cells loaded to the left well through tilting the chip system. After returning chip to the horizontal position, *Syntrophomonas wolfei* were loaded and the coculture was further incubated for over 20 hours.

**Supplementary Video 3.** The cell movement of a single identical organism in the microfluidic system. Same microfluidic operation as shown in Video 1 was conducted except that a single identical organism, *Syntrophomonas wolfei*, was loaded twice. Here, after loading more cells of *Syntrophomonas wolfei* to the right side well and returning chip to the horizontal position, *Syntrophomonas wolfei* were loaded second time. No further incubation was conducted as *Syntrophomonas wolfei* alone cannot grow on butyrate contained in the flow media.

**Figure S1**


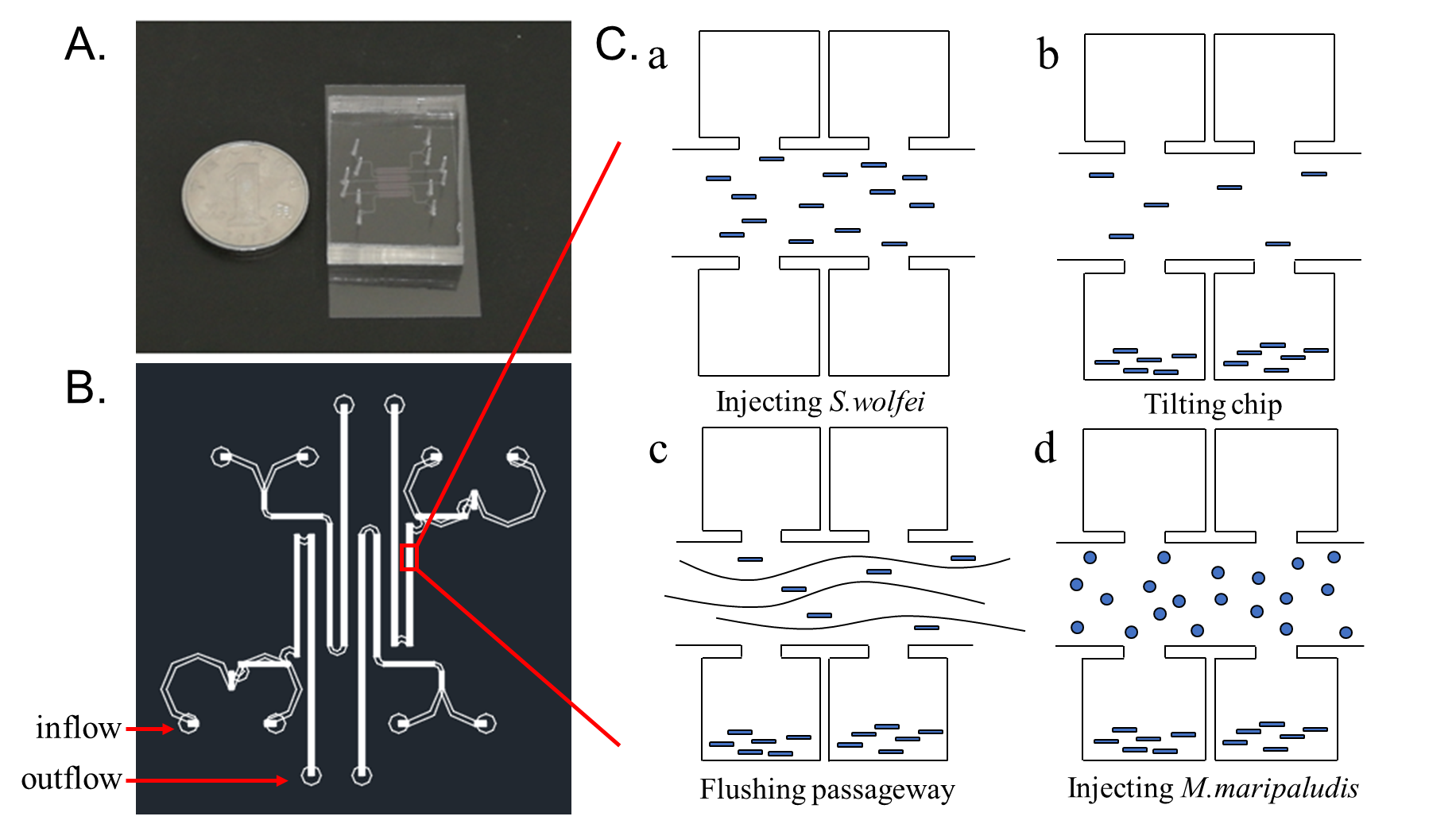


**Supplementary Figure 1**. Microfluidic chip and experimental setup. (A) overview of chip system with the coin shown for the comparison of chip size; (B) a schematics of the device. The device was composed of four identical units. There are two inflows and one outflow to each unit; (C) experimental setup showing (a) the loading of the first organism, (b) tilting the chip so that more cells were located to the one side wells, (c) washing out the remaining cells in the chip channels, and (d) loading of the second organism.

**
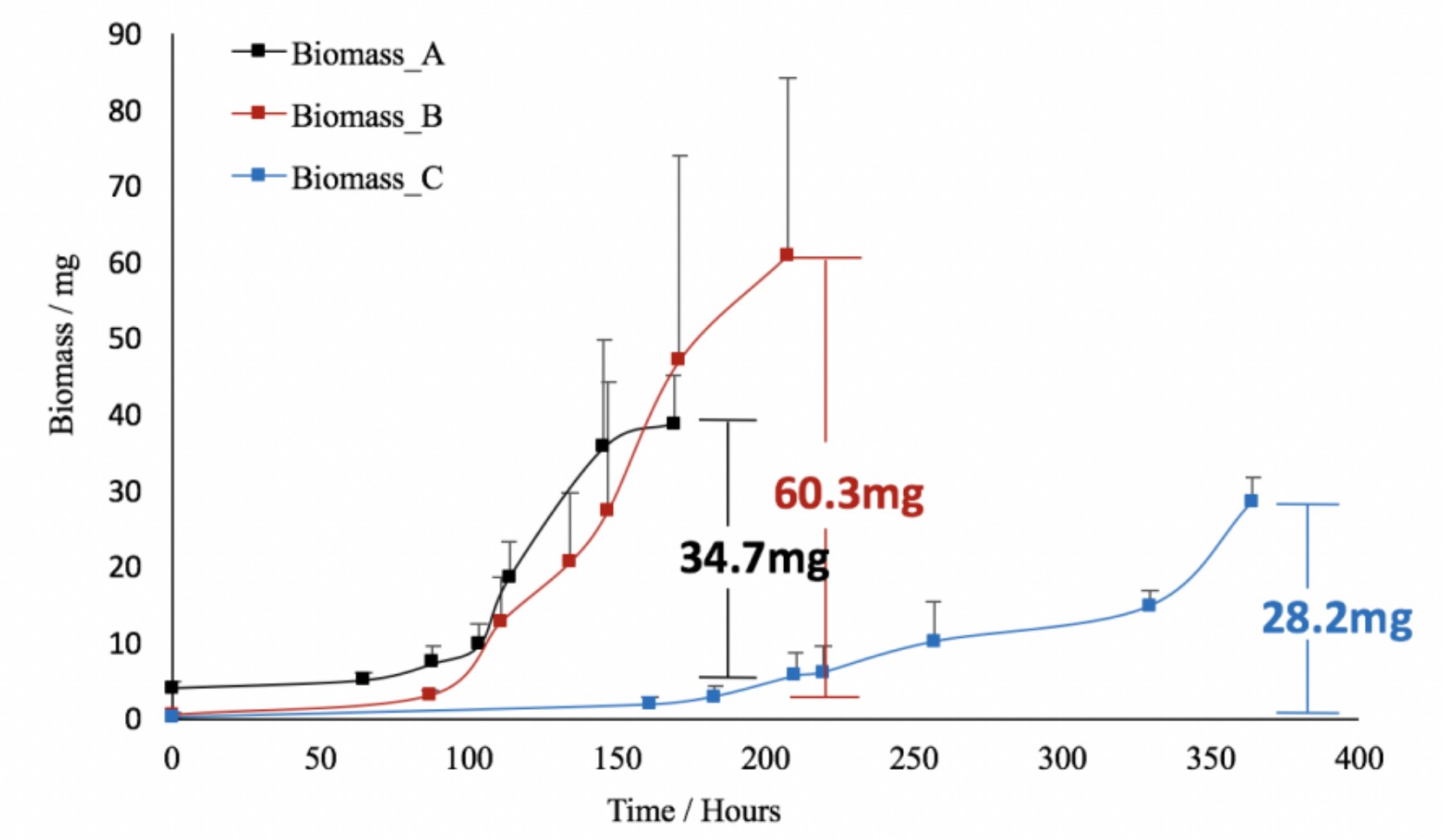
Figure S2**

**Supplementary Figure 2:** The total biomass production of a thermophilic coculture consisting of *Syntrophothermus lipocalidus* and *Methanocella conradii* estimated by qPCR of subsamples. The actively growing *S. lipocalidus* and *M. conradii* in pure cultures were inoculated to fresh medium with different population sizes so that three levels of syntroph-to-methanogen cell ratios (R) were created at the beginning for cocultures. Biomass_A, R=24:1; Biomass_B, R=3:1; Biomass_C, R=1:3. The error bars indicate the positive values of standard deviation of four replicates. The data indicate the net accumulation of biomass from three different treatments.


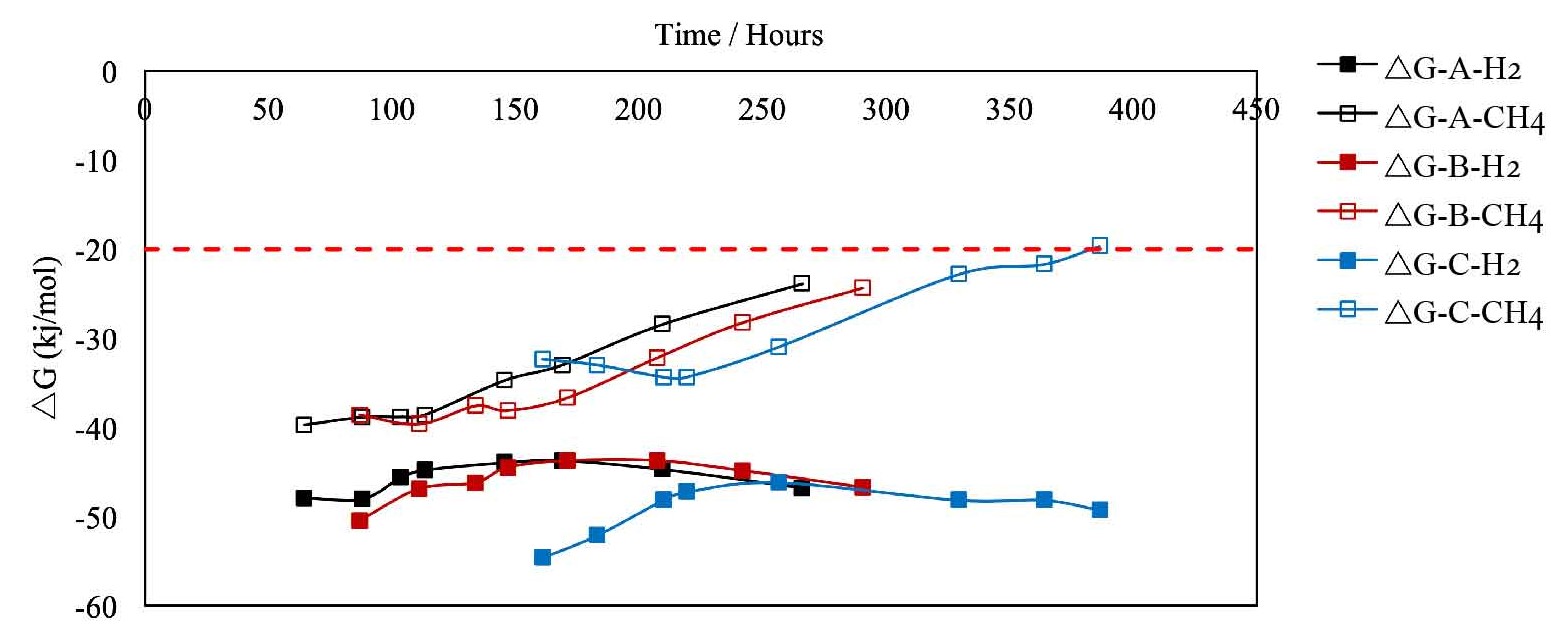
**Figure S3**

**Figure S3:** Gibbs free energy change available for *Syntrophothermus lipocalidus* and *Methanocella conradii* during their syntrophic growth on butyrate with different treatments of the initial cell ratio (R). Gibbs free energies (ΔG) of the individual reactions were calculated from the standard Gibbs free energies (ΔG^o^) and the actual concentrations of reactants and products as described previously.The standard Gibbs free energies (ΔG^o^) of the reactions were calculated from the standard Gibbs free energies of formation (G_f_^o^) of the reactants and products using literature data. A, R=24:1; B, R=3:1; C, R=1:3. Closed and open symbols indicate the Gibbs free energies of the butyrate-oxidizing and H_2_-producing reaction (H_2_) and the H_2_-oxidiang and CH_4_-producing reaction (CH_4_), respectively.

**References:**

Rothfuss, F., and Conrad, R. (1993). Thermodynamics of methangenic intermediary metabolism in littoral sediment in littoral sediment of Lake Constance. *Fems Microbiology Ecology* 12(4)**,** 265-276. doi: 10.1111/j.1574-6941.1993.tb00039.x.

Thauer, R.K., Jungermann, K., and Decker, K. (1977). Energy-conservation in chemotropic anaerobic bacteria. *Bacteriological Reviews* 41(1)**,** 100-180. doi: 10.1128/mmbr.41.1.100-180.1977.
